# Mucinous tumors arising from ovarian teratomas as another source of pseudomyxoma peritoneii: MR findings comparison with ovarian metastases from appendiceal mucinous tumors

**DOI:** 10.1259/bjro.20220036

**Published:** 2023-04-19

**Authors:** Yumiko Oishi Tanaka, Emiko Sugawara, Akiko Tonooka, Tsukasa Saida, Akiko Sakata, Yosuke Fukunaga, Hiroyuki Kanao, Toyomi Satoh, Masayuki Noguchi, Takashi Terauchi

**Affiliations:** 1 Diagnostic Imaging Department, Cancer Institute Hospital of Japanese Foundation for Cancer Research, Tokyo, Japan; 2 Department of Diagnostic & Interventional Radiology, Tsukuba University Hospital, Tsukuba, Japan; 3 Department of Pathology, Cancer Institute Hospital of Japanese Foundation for Cancer Research, Tokyo, Japan; 4 Division of Pathology, Cancer Institute, Japanese Foundation for Cancer Research, Tokyo, Japan; 5 Department of Diagnostic & Interventional Radiology, Faculty of Medicine, University of Tsukuba, Tsukuba, Japan; 6 Department of Diagnostic Pathology, Tsukuba University Hospital, Tsukuba, Japan; 7 Colorectal Surgery Department, Cancer Institute Hospital of Japanese Foundation for Cancer Research, Tokyo, Japan; 8 Department of Gynecologic Oncology, Cancer Institute Hospital of Japanese Foundation for Cancer Research, Tokyo, Japan; 9 Department of Obstetrics & Gynecology, Faculty of Medicine, University of Tsukuba, Tsukuba, Japan; 10 Department of Diagnostic Pathology, Faculty of Medicine, University of Tsukuba, Tsukuba, Japan

## Abstract

**Objective:**

The origin of pseudomyxoma peritoneii (PMP) has been established as low-grade appendiceal mucinous tumors (AMT). However, intestinal-type ovarian mucinous tumors are known as another source of PMP. Recently, it is advocated that ovarian mucinous tumors causing PMP originates from teratomas. However, AMTs are often too small to detect on imaging; then, differentiating metastatic ovarian tumors of AMT from ovarian teratoma-associated mucinous tumors (OTAMT) is important. Therefore, this study investigates the MR characteristics of OTAMT compared to the ovarian metastasis of AMT.

**Methods:**

MR findings of six pathologically confirmed OTAMT were retrospectively analyzed compared to ovarian metastases of low-grade appendiceal mucinous neoplasms (LAMN). We studied the existence of PMP, uni- or bilateral disease, the maximum diameter of ovarian masses, the number of loculi, a variety of sizes and signal intensity of each content, the existence of the solid part, fat, calcification within the mass, and appendiceal diameters. All the findings were statistically analyzed using the Mann–Whitney test.

**Results:**

Four of the six OTAMT showed PMP. OTAMT showed unilateral disease, had a larger diameter, more frequent intratumoral fat, smaller appendiceal diameter than those in AMT, and they were statistically significant (*p* < .05). On the other hand, the number, variety of size, signal intensity of loculi, and the solid part, calcification within the mass did not differ from each other.

**Conclusion:**

Both OTAMT and ovarian metastasis of AMT appeared as multilocular cystic masses with relatively uniform signal and size of loculi. However, a larger unilateral disease with intratumoral fat and smaller size of the appendix may suggest OTAMT.

**Advances in knowledge:**

OTAMT can be another source of PMP, as AMT. MR characteristics of OTAMT were very similar to ovarian metastases of AMT; however, in cases with PMP combined with fat-containing multilocular cystic ovarian mass, we can diagnose them as OTAMT, not PMP caused by AMT.

## Introduction

Pseudomyxoma peritoneii (PMP) is defined as grossly evident peritoneal involvement of the jelly-like mucoid.^1–3^ Ovarian tumors with abundant mucinous material were often complicated with PMP.^4^ However, clinicopathological features,^5^ immunohistochemical staining,^6^ and genetic predisposition^7^ have revealed that PMP usually originates from low-grade appendiceal mucinous neoplasm (LAMNs).^8^ Conversely, PMP is rarely caused by ovarian mucinous tumors of the germ cell origin^8–10^ and usually appears as a mature teratoma component. The histological characteristics of ovarian teratoma-associated mucinous tumors (OTAMTs) are more similar to those of appendiceal or intestinal mucinous neoplasms than those of primary ovarian mucinous neoplasms.^9^ Therefore, OTAMTs would be misdiagnosed as metastatic ovarian tumors from primary appendiceal mucinous tumors (AMTs) even after histopathological diagnosis. Usually, a primary AMT is treated by appendectomy, ileocecal resection, or right hemicolectomy.^11^ On the other hand, a primary borderline or malignant ovarian tumor is treated with total hysterectomy, bilateral salpingo-oophorectomy, omentectomy, and optimal debulking of the intraperitoneal dissemination. Differentiation of these two disease entities with pre-operative imaging diagnosis may play some roles in choosing the treatment of choice. Recent studies have fully clarified imaging findings of primary mucinous ovarian neoplasms^12,13^ and metastatic ovarian tumors of intestinal origin.^13–15^ However, imaging characteristics of OTAMTs have not been reported to the best of our knowledge. Therefore, this study aimed to investigate MR characteristics of OTAMTs compared to those of AMTs, including LAMNs and mucinous adenocarcinomas with PMP and ovarian metastasis.

## Methods

### Patients

From April 2013 to September 2020, we found six cases of histopathologically proven OTAMTs with pre-operative MR examination after a careful search of the MR database of our institutes. These six consecutive patients were included in this study. During the same period, primary appendiceal tumors diagnosed with CT and/or MR at Cancer Institute Hospitalwere also depicted in the CT/MR database as a potential control group. After excluding the patients without MR (*n* = 2), ovarian masses (*n* = 4), and non-mucinous pathology (*n* = 6), six AMTs with ovarian metastasis were included as a control group.

The histopathological diagnosis was based on institutional pathology reports. In addition, we confirmed histopathological diagnosis of OTAMTs using immunohistochemical staining including CK7, CK20 and searching the teratomatous components within the tumors. The histopathological diagnosis of OTAMT was mucinous adenocarcinoma, borderline mucinous tumor, and mucinous cystadenoma in one, four, and one patient, respectively. The AMT group included an adenocarcinoma with neuroendocrine differentiation and partly LAMN component, a partially mucinous adenocarcinoma in adenoma, a mucinous adenocarcinoma with LAMN, and three LAMNs. Patients' detailed data are shown in Table 1.

**Table 1.** Summary of the cases

	Age	Purpose of exam	Surgery	Pathological diagnosis			
				Ovary		Appendix	Pseudomyxoma peritoneii
				Mucinous tumor	Teratoma		
OTAMT	67	Ovarian tumor	TAH + BSO + pOM	Carcinoma	Mature	NA	None
	20	Ovarian tumor	LSO R Cystectomy Appendectomy Omental biopsy	Borderline	Mature	Normal	DPAM
	19	Ovarian tumor	RSO Omental biopsy LNs biopsy	Borderline	Mature	NA	None
	40	Ovarian tumor	TAH + RSO + pOM	Borderline	Mature	NA	DPAM
	53	Ovarian tumor	BSO + TAH + pOM	Borderline	Mature	NA	Mucin without epithelial cells
	62	Ovarian tumor	BSO	Adenoma	Mature	NA	Mucin without epithelial cells
AMT	53	Ovarian tumor	ICR + BSO + OM	Adenocarcinoma	None	Adenocarcinoma with partial LAMN component	PMCA
	61	Ovarian tumor	Appendectomy + BSO	Adenocarcinoma	None	Adenocarcinoma with adenoma	PMCA
	57	Ovarian tumor	ICR + BSO + OM Cholecystectomy Splenectomy	Adenocarinoma	None	Adenocarinoma	PMCA
	52	Ovarian tumor	ICR + BSO + OM Supravaginal amputation Cholecystectomy Splenectomy	LAMN	None	LAMN	DPAM
	48	Ovarian tumor	ICR + BSO + OM	LAMN	None	LAMN	DPAM
	39	Psedomyxoma peritoneii	ICR + BSO + OM Supravaginal amputation Cholecystectomy Splenectomy	LAMN	None	LAMN	DPAM

AMT, appendiceal mucinous tumor; DPAM, disseminated peritoneal adenomucinosis; ICR, ileocecal resection; NA, not applicable; OM, omentectomy; OTAMT, ovarian teratoma-associated mucinous tumors; PMCA, peritoneal mucinous carcinomatosis; SO, salpingo-oophorectomy; TAH, total abdominal hysterectomy.

### MR examinations and supplemental imaging modalities

MR examinations were obtained using variable equipment, including 1.5 T (*n* = 8) and 3T (*n* = 4) magnetic field strength. Sagittal and axial or coronal T1-, T2-, fat-saturated T1-, and diffusion-weighted images (*T*_1_WI, *T*_2_WI, FS*T*_1_WI, and DWI) using a b value of 800–1000 s/m^2^ were obtained in all patients with 280–400 mm of a field of view and a 4–8 mm slice thickness with a 0–4 mm intersection gap. Contrast enhancement was available in all but one case. Contrast CT (a 1–5 mm slice thickness and interval) was available in 10 cases, and ^16^F-fluorodeoxyglucose-positron emission tomography-CT (attenuation-correcting CT with a 6.52 slice thickness and gap) was supplementally performed in one case instead of CT.

### Evaluation of MR examinations and supplemental imaging modalities

All MR images were retrospectively analyzed by a board-certificated radiologist who devoted professional attention to gynecologic imaging for more than 20 years blinded to the pathologic diagnosis. We evaluated whether the ovarian mass is unilateral or bilateral and assessed the maximum diameter of the masses. We measured the diameter of the larger masses when bilateral diseases. As all the masses were multilocular cystic masses, we counted the number of loculi, and measured size ratio of the largest to smallest loculus, signal ratio of the darkest to brightest loculus on *T*_2_WI, and standard deviation of the signal of the loculi on *T*_2_WI of larger ovarian masses. These parameters were calculated as it had been reported that they are significantly different from each other between primary and metastatic ovarian tumors.^13^ The existence of intratumoral fat and calcification was also noted in all ovarian groups. Appendices were identified on *T*_2_WI supported by supplemental CT. We measured the maximum diameter of the appendix on *T*_2_WI and evaluated as enlarged when the maximum diameter was larger than 6 mm. We evaluated it as PMP only when a large number of ascites with mass effects, such as the deformity of the uterus, eccentric deviation of the intestines, or uneven distribution of the ascites, were noted.

### Statistical analysis

All the parameters in AMT and OTAMT were analyzed using the Mann–Whitney *U* test and considered statistically significant when the *p*-value was <0.05.

### Ethics approval and consent to participate

The institutional review board approved this retrospective study of our institutes (2021–1007 for Cancer Institute Hospital, R03-052 for University of Tsukuba) with a waiver of informed consent. Study procedures were carried out under the Declaration of Helsinki and the Good Clinical Practice guidelines.

## Results

The results are presented in Table 2.

**Table 2.** Imaging findings of OTAMTs^*a*^ & AMTs^*b*^

			OTAMT	AMT	Statistical analysis
Patients’ age		Median	46.5	52.5	N.S.
Ovarian mass	Bilateral disease	Bilateral	0	4	N.S.
		Unilateral	6	2	
	Maximum diameter (cm)	Median	23.85	11.65	*p* < 0.05
	Number of loculi	Median	96.5	54	N.S.
	Number of loculi/Maximum diamter	Median	4.035	5.014	N.S.
	Size ratio of loculi	Median	111.2	63.44	N.S
	Signal ratio of loculi	Median	14.94	8.754	N.S
	Standard deviation of signal	Median	558.7	219.6	N.S
	Solid part (number of cases)	Yes	2	2	N.S
		No	4	4	
	Intratumoral fat (number of cases)	Yes	5	0	*p* < 0.01
		No	1	6	
	Intratumoral calcification (number of cases)	Yes	3	0	N.S
		No	3	6	
Appendiceal diameter (cm)		Median	5.5	16.05	*p* < 0.01
Pseudomyxoma peritoneii (number of cases)		Yes	4	6	N.S
		No	2	0	

AMT, appendiceal mucinous tumor; OTAMT, ovarian teratoma-associated mucinous tumors.

a Ovarian teratoma-associated mucinous tumors.

b Appendiceal mucinous tumors.

**Figure 1.**
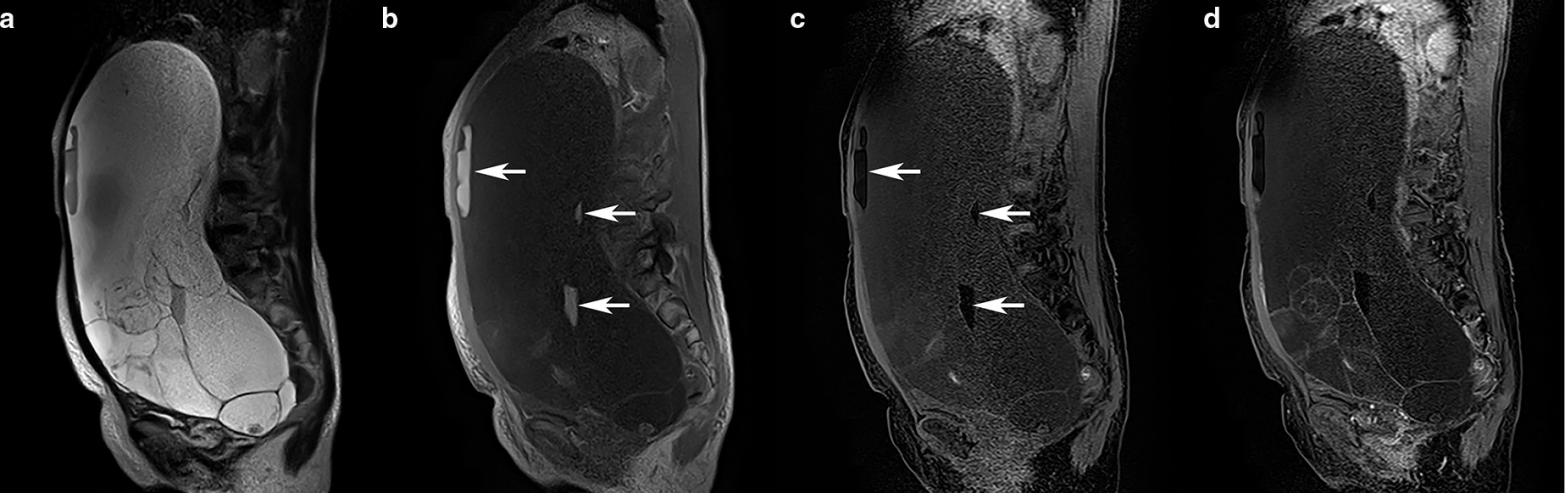
53-year-old female with a borderline mucinous tumor with mature teratoma. A huge multilocular cystic mass with fluid levels on sagittal *T*_2_ weighted images (**a**) widely occupied the peritoneal cavity. The upper part of the fluid level shows high-signal intensity on *T*_1_ weighted images (**b**, arrows) and signal suppression on fat-saturated *T*_1_ weighted images (**c**, arrows). Thin septa and capsules were enhanced after administering contrast materials, but no solid parts were identified (**d**). The left ovary was normal, and the maximum diameter of the appendix was 3 mm (not shown). The mass was suspected as the right ovarian origin on MR, which was confirmed histopathologically.

**Figure 2.**
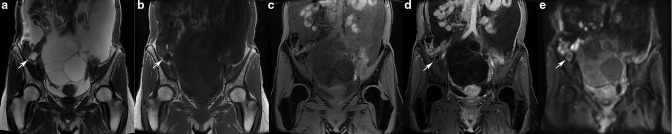
52-year-old female with low-grade mucinous appendiceal neoplasm with left ovarian metastasis. A blind-ended tubular mass with a maximum diameter of 16.8 mm was demonstrated on coronal *T*_2_ weighted images (**a**, arrow). Coronal T2-, T1- (**b**), fat-saturated T1- (**c**), and contrast-enhanced and fat-saturated *T*_1_ weighted images (**d**) also demonstrate a multilocular cystic mass attached to the left pelvic wall and a large amount of ascites. These ascites show a higher signal than urine in the bladder (not shown in figures) on diffusion-weighted images (e, *b* = 1000 s/m^2^), indicating viscous fluid. The right ovary was normal, and the mass is suspected as the left ovarian origin on MR, which was confirmed histopathologically.

The OTAMTs (Figure 1) were significantly larger, including fats within the ovarian mass more frequently than in the ovarian metastases of AMTs. Conversely, the appendiceal maximum diameter was larger than 10 mm in all cases with AMTs, but none in OTAMTs (Supplementary Table 1). the diameter of the appendix was larger in AMTs (Figure 2). All other parameters, including different sizes, the signal intensity of each loculus, solid part, or calcification in the ovarian masses, did not show a statistically significant difference.

## Discussion

The term PMP has existed in the literature for more than 100 years, yet there is no consensus on its definition.^1,2^ PMP has been used simply for the macroscopic appearance of mucinous ascites and not as a histologic diagnosis but in the absence of an alternative terminology widely accepted.^3^ Ronnett et al classified PMP into disseminated peritoneal adenomucinosis (DPAM) and peritoneal mucinosis (PMCA) based on the degree of cytologic atypia and mitotic activity of the extracellular mucin rather than the primary tumor.^2^ Furthermore, a condition in which mucin without epithelial cells is distributed in the peritoneal cavity has also been reported.^2^

The primary ovarian mucinous borderline tumors have been believed as the source of PMP for a long time,^4^ as ovarian tumors with abundant mucinous material—the so-called pseudomyxoma ovarii—were often complicated with PMP. However, clinicopathological features,^5^ immunohistochemical staining,^6^ and genetic predisposition^7^ have revealed that PMP usually originates from LAMNs.^8,10^ Because of the high rate of ovarian metastases^11^ and difficulty in diagnosing LAMNs pre-operatively, many misdiagnosed cases are believed to originate from primary ovarian mucinous neoplasms. Most primary ovarian mucinous neoplasms are developed from mucinous cystadenoma, in other words, surface epithelial origin. However, primary mucinous tumors originating from ovarian teratoma and Brenner tumors are also noted,^16^ Recently, PMP is also caused by ovarian mucinous tumors of the germ cell origin.^8,17^ It usually appears as a component of mature teratoma, and its histological characteristics are similar to those of appendiceal or intestinal mucinous neoplasms rather than primary ovarian mucinous neoplasms.^9^

Imaging findings of primary mucinous ovarian neoplasms had shown multilocular cystic mass with variable size and signal intensity of the loculi^12^ —the so-called stained glass appearance.^13^ The metastatic ovarian mass has been reported to show multilocular cystic masses^15,18^ with more uniform size and signal of each loculus on MR.^13^ Then, the radiologists should consider the possibility of metastatic ovarian tumors and search the primary site; however, appendiceal masses are often missing because of their small size. In this study, all ovarian masses of the OTAMTs and ovarian metastases of the AMTs showed multilocular cystic masses. Then, we compared the variation of size and signal of the loculi in each group, but neither offered a significant difference. It may be because the mucinous tumor of germ cell origin is biologically equivalent to the mucinous tumor of intestinal origin (*i.e.* the immunohistochemical stain results, including CK7, CK20, and CDX2).^9^ Therefore, both conditions have very similar MR characteristics and only differ due to reported primary mucinous tumors—the so-called stained glass appearance.^12,19^

Ovarian teratomas are composed of various tissues derived from all three germ cell layers. However, mature teratomas are usually encapsulated by squamous cell epithelium with accessory glands, such as the hair follicle, sweat, and sebaceous glands.^20^ Then, mature teratoma usually includes sebaceous fat, hair, and keratinized materials. Fat tissue is a hallmark of ovarian teratoma in imaging diagnosis.^21,22^ Fat tissue in OTAMT also seemed to be a pathognomonic MR finding of each disease, and MR could reveal those in all cases in this study. However, a small amount of fat was missed during the initial pathological examination in our two cases. The mean diameter of OTAMT might be too large for a detailed pathological investigation. Imaging modalities, including CT or MR, can easily detect a small amount of fat for multisectional capability. Therefore, diagnostic radiologists should carefully investigate the signal intensity on MR or density on CT of the contents of multilocular cystic masses to depict a small amount of fat and inform the diagnostic pathologists pre-operatively.

AMT is typically characterized as a centrally hypoattenuating blind-ending tubular structure in imaging examinations, with wall enhancement contiguous with the cecum. However, prospective imaging diagnosis is often challenging, especially in LAMNs, because the mucous-filled appendix, known as mucocele, often results in mucosal inversion, intussusception, and rupture.^23^ Although ruptured appendiceal mucocele might be hardly depicted with neither CT nor MR, careful observation around the cecum enabled its detection in our present series.

Imaging findings of PMP have been reported using CT^24,25^ and MRI,^26,27^ including intraperitoneal cystic mass or loculated ascites with thin capsules, septations or calcification, scalloping of the liver margins, bowel loop displacement, omentum infiltration, and visceral organ invasion. Sometimes, the fluid shows a high signal on proton density images^26^ and restricted diffusion.^27^ Among them, scalloping of the solid organ may be a key finding differentiating PMP from ascites. Recently, Hotta et al reported that visceral scalloping on CT is a predictor of recurrence after a complete cytoreductive surgery.^28^ In our study with MR, the signal intensity of the ascites could have been evaluated; however, we could not compare the signal intensity or ADCs due to the heterogeneity of the used equipment and sequences. Visceral scalloping was assessed as the uterus deformity and helped identify the PMP and deformity or eccentric deviation of the intestines. Bechtold et al reported that large volumes of mucinous ascites and calcification in the masses are more common in DPAM, and visualization of a primary mass or peritoneal implant was common in the PMCA on CT.^29^ We did not evaluate the detailed characteristics of ascites; however, we considered them PMP because of uneven distribution. In our cases, only two cases of OTAMTs showed DPAM, and another two showed mucin without epithelial cells. In contrast, AMT was complicated with PMCA in three and DPAM in the remaining three. Although OTAMT has been increasingly noticed as another source of PMP, its incidence could be lower than that of AMTs. Therefore, further investigation should be performed to verify our conclusion.

Ovarian masses in our study were too large to investigate precisely on histopathological examination, which might result in missing more cases during the study period. We found two cases with OTAMTs during the second inspection to determine a small amount of fat, based on the imaging finding after approving the first pathological diagnosis reports. This fact shows the possibility of many missing cases during the study period, although OTAMT is a rare disease entity. As our results of the present study may be able to depict more cases with OTAMTs, we would like to re-evaluate the imaging characteristics of OTAMTs with more numbers of cases near future,

Our series was collected in two institutes with a long study period and with varied equipment and sequences, and retrospectively evaluated which might be a limitation of this study. Another limitation of this study is the small number of cases. Metastatic ovarian tumors of appendiceal origin may not be so rare; however, pathologically proven OTAMTs are merely reported. Therefore, we could find out only six cases with OTAMTs in this study period. It may be a limitation of this study. Histopathological examination of the appendix was performed in only one case with OTAMTs as the appendix seemed normal during the surgery. This may be the third limitation of our study.

Although PMP is usually caused by LAMNs that often cause ovarian metastases, OTAMT is another source of PMP. Our results showed that MR characteristics of OTAMTs were very similar to ovarian metastases of AMTs; however, the maximum diameter was larger than the AMT’s metastases, the mass included fat tissues, and no enlarged appendix was noted. In cases with multilocular cystic ovarian mass with viscous ascites indicating PMP, radiologists should carefully identify small amounts of fat within the mass and enlarged appendix, pathognomonics of OTAMTs and ovarian metastasis of the AMTs, respectively.
